# Biomarkers of Hand Osteoarthritis Are Detectable after Mechanical Exercise

**DOI:** 10.3390/jcm8101545

**Published:** 2019-09-26

**Authors:** Anna Bender, Ulrich Kaesser, Gerrit Eichner, Georg Bachmann, Juergen Steinmeyer

**Affiliations:** 1 Laboratory for Experimental Orthopaedics, Department of Orthopaedics, Justus Liebig University Giessen, Paul-Meimberg-Str. 3, 35392 Giessen, Germany; 2 Internistisches Praxiszentrum, 35392 Giessen, Germany; 3 Mathematical Institute, Justus Liebig University Giessen, 35392 Giessen, Germany; 4 Georg Bachmann, Department of Diagnostic Radiology, Kerckhoff-Klinik GmbH, 61231 Bad Nauheim, Germany

**Keywords:** biomarker, serum, hand osteoarthritis, exercise, PIIANP, CPII, COMP

## Abstract

Background: Hand osteoarthritis (OA) is one of the most common joint diseases, but studies on biomarkers are rare. The aim of this explorative study was (a) to evaluate potential biomarkers of hand OA, (b) to identify an optimal time point to sample venous blood, and (c) to correlate biomarker levels with radiological and clinical scores. Methods: Four female cohorts were investigated. One with a more Heberden-accentuated OA and one with a more Bouchard-accentuated hand OA, and two symptom-free control groups aged 20–30 or 50–75 years. The venous blood was sampled before and at eight time points after mechanical exercise of the OA hand. X-rays of OA hands were assessed using the Kellgren and Lawrence as well as Kallman scores. Participants were evaluated clinically using the AUSCAN™ Index, visual analog scale (VAS), and Health Assessment Questionnaire (HAQ). Serum levels of seven biomarkers were measured by ELISA. Results. The concentrations of CPII, COMP, IL-15, sVCAM-1, NGAL, and PIIANP were significantly increased within 15 min after exercise. PIIANP was markedly elevated in the Heberden-accentuated OA group as compared to both control groups, but did not correlate with any radiological or clinical score. Analysis of the probabilistic index further revealed that CPII can distinguish between Bouchard’s OA and premenopausal controls whereas COMP can discriminate between Bouchard’s and Heberden’s OA. Conclusions: This study demonstrates that even previously undetectable biomarkers can be quantified in serum after mechanical exercise. Future larger studies are needed to determine specificity and sensitivity of these markers and their ability to diagnose even pre-radiological OA.

## 1. Introduction

Hand joints have been reported to have the highest prevalence for osteoarthritis (OA) compared to hip and knee joints [1,2,3]. The lifetime risk of developing symptomatic hand OA is nearly 40% [4]. Several studies found an age-dependent prevalence of radiographically confirmed and symptomatic hand OA especially affecting the distal interphalangeal (DIP) joints, followed by the first carpometacarpal (CMC1), the proximal interphalangeal (PIP), and finally and more rarely the metacarpophalangeal (MCP) joints [1,5,6,7]. About one third of patients with OA of the DIP joints also develop OA of the PIP joints. Heberden’s and Bouchard’s nodes as well as OA of the CMC1 or trapezio-scaphoid (TS) joint are typical variants of hand OA. This joint disease is clinically characterized by pain, stiffness, increased sensitivity to cold, disability, joint swelling, and ultimately even deformation of the joint. Women are more often affected by OA, with no preference for either hand [1,5,6,7]. Hand OA may occur alone without any OA diagnosed in larger joints and is considered to be a multifactorial complex disorder of the whole joint [8,9,10,11,12,13]. Since research has focused mainly on large joints, our knowledge about hand OA is limited and is derived mainly from knee OA.

Diagnosis of hand OA is mostly performed by physical examination and X-ray imaging. Currently, no standard blood or urine biomarker is available and validated for early diagnosis, staging, or predicting outcomes in clinical trials. Studies on biomarkers of hand OA are rare. Analysis of blood is hampered by background levels of biomarkers derived from other OA joints, especially from the larger hip, knee joints, and the spine. Due to the small size of the finger joints, analysis of the synovial fluid would be more useful, but is practically difficult.

The few studies on the levels of potential biomarkers found them to be only slightly elevated in the serum of patients with hand OA [14,15,16,17,18,19]. Increased serum levels of the cartilage collagen type II cleavage neoepitope Col2-3/4short [15], soluble vascular cell adhesion molecule-1 (sVCAM-1) [16], hyaluronic acid (HA), and cartilage oligomeric matrix protein (COMP) [14,18,20] were determined in patients with hand OA, whereas other markers, including the collagenous neoepitope C2C, chondroitin sulphate 846 (CS846) epitope [15], and two growth factors [16] remained unchanged.

Remarkably, associations between biomarkers and radiographic scores as well as clinical criteria of hand OA were observed in two larger studies [17,18]. Pantsulaia et al. [17] reported that of the seven markers investigated, only osteoprotegerin (OPG) correlated with the radiological Kellgren and Lawrence (KL) score as well as with the number of affected finger joints, suggesting that OPG might be a valid biomarker for hand OA. Hand OA was diagnosed using various physical examinations including the American College of Rheumatology (ACR) criteria [21], and associations with some OA biomarkers such as HA and COMP were found [18]. However, whether biomarkers also correlate with scores specifically developed to evaluate radiologically discernible alterations of hand OA such as the Kallman score [22,23], or whether they correlate with pain, stiffness, and physical function of OA-affected hands according to measures such as the Australian/Canadian (AUSCAN™) Index [24,25,26,27,28], remains to be investigated. 

Mechanical stimulation of joints appears to have an impact on the level of possible biomarkers and thus on the optimal time point for sampling [23,24,25,26,27,28,29,30,31,32]. In particular, elevated serum levels of COMP were found within 30 min after a predefined exercise in patients with OA knee joints [29], physically active healthy adults [30], and marathon runners [31]. This time period (30 min) appears to represent the time it takes for COMP and probably other cartilage biomarkers to diffuse from larger joints into the venous blood. These studies also underscore the role of exercise as a potential confounding factor in biomarker studies. 

Diagnostic and prognostic biomarkers of early stages of OA would enable prophylactic and therapeutic measures to be deployed much earlier than is currently possible. This may restrict or even prevent disease progression and joint replacement surgeries, and preserve a joint mobility which is free of pain. In this study, we tested the hypothesis that mechanical stimulation of OA-afflicted hands stimulates the release and diffusion of biomarkers from cartilage and synovial fluid to venous blood and in doing so makes them quantifiable. We examined whether the levels of seven OA biomarkers are also elevated in patients with hand OA as compared to healthy controls. Furthermore, we also determined whether elevated biomarkers correlate with various radiographical and clinical scores that were specifically developed to evaluate hand OA. 

Using our new approach to mechanically stimulate the release and diffusion of biomarkers from hand and finger joints to venous blood, we quantified previously undetectable biomarkers, observed marked differences between the different cohorts, and calculated possible correlations with clinical and radiological scores.

## 2. Methods

### 2.1. Patients—Inclusion and Exclusion Criteria 

The serum originated from 12 premenopausal controls (“pre-group”), 12 postmenopausal test subjects (“post-group”), and 24 postmenopausal patients with hand OA (“OA-group”). All subjects gave their informed consent for inclusion before they participated in the study. The study was conducted in accordance with the Declaration of Helsinki, and the protocol was approved by the Ethics Committee of the Faculty of Medicine (Justus Liebig University Giessen, Giessen, Germany; Az. 145/08). We used the ACR criteria [21] to subcategorize the OA changes seen in the hand joints thereby diagnosing 12 female patients with a more Heberden and 12 patients with a more Bouchard-accentuated hand OA (Table 1). Furthermore, every finger joint (DIP, PIP, IP1, MCP, und CMC1) was examined physically, and by digital X-raying of the index hands. The index hand was defined as the hand being most affected by OA symptoms for the patient group, whereas one hand was randomly selected as the index hand for both control groups. Table 1 reveals that the two OA sub-cohorts did not differ with respect to age, BMI, CRP, AUSCAN™ scores, and VAS pain. Table 1 illustrates that none of the pre- or postmenopausal controls had any symptoms of hand OA or macroscopically visible nodes. However, we did not take any X-rays of the healthy pre- and postmenopausal controls for ethical and legal reasons. 

Only adults who fulfilled the inclusion criteria were recruited in the order of their appearance. Probands needed to be females and should have had a body-mass-index (BMI) between 18 and 30 kg/m^2^. The patient group (“OA-group”) as well as one control group (“post-group”) was postmenopausal and aged between 50 and 75 years. The third cohort (“pre-group”) was considered to be premenopausal and joint healthy due to the selected age which ranged from 20 to 30 years. The main exclusion criteria were the presence of other inflammatory, rheumatic or degenerative diseases, Dupuytren’s contracture, metabolic, kidney or liver diseases, fractures, surgery during the preceding 24 months, tumor/cancer, mental disorders, drug abuse, immunodeficiency, and treatment with corticosteroids or hyaluronate or bisphosphonates. Non-steroidal anti-inflammatory drugs (NSAIDs) and other analgesics were discontinued 7 days prior to entering the study. Paracetamol up to 3 × 500 mg was allowed as a rescue medication and was taken by three patients. 

### 2.2. Study Performance

At the beginning of our exploratory study, all participants were asked to fill out the AUSCAN™ Index questionnaire [24,25], the Health Assessment Questionnaire (HAQ) [30,31], and the visual analog scale for pain (VAS pain). The proprietary AUSCAN™ Index is a validated score which uses a self-administered questionnaire to specifically evaluate pain, stiffness, and basic life skills in patients with hand OA. The HAQ estimates the daily impairment of normal life induced by a joint disease, where eight subareas of daily activities are independently scored by the patients from 0–3 [33,34]. Additionally, hand pain was solely scored by the participants using a 100 mm VAS ranging from no to extreme pain.

This was followed by fasting venous blood collection in the morning from the forearm of the index hand using a peripheral venous line. The blood samples were taken before and 1, 2, 5, 10, 15, 30, 45, and 60 min after exercise of the index hand. The level of exercise was controlled by compressing a blood pressure cuff inflated to 30 mm Hg 20 times with the index hand. Blood samples were centrifuged at 2000 g for 10 min, aliquoted and stored at −80 °C until analyses.

The radiographs of the index hand were only taken from patients, with 11 finger joints (DIP, PIP, IP1, CMC1, and TS) of the index hand being scored according to KL [7,35] and Kallman [22,23]. The maximum possible KL score was 60 per hand. The Kallman score evaluates six radiologically discernible OA criteria such as the size of osteophytes, joint space narrowing, the presence or absence of subchondral sclerosis and cysts, and the destruction and deformation of the joint [22,23]. The maximum possible Kallman-Score was 104 per hand.

### 2.3. Biomarker Analysis

The following biomarkers were studied: (1) A marker of cartilage synthesis in the form of C-terminal propeptide of procollagen type II (CPII) and N-terminal propeptide of type II procollagen (PIIANP), (2) a marker of cartilage resorption in the form of collagen type II ¾ cleavage product (C1,2C) and cartilage oligomeric matrix protein (COMP), and (3) inflammatory markers such as neutrophil-gelatinase-associated lipocalin (NGAL), interleukin-15 (IL-15), and soluble-vascular-cell-adhesion-molecule-1 (sVCAM-1). We used the commercially available enzyme-linked immunosorbent assay (ELISA) kits to determine these seven serum biomarkers, namely CPII (IBEX Technologies Inc.), COMP (Euro-Diagnostica) C1,2C (IBEX Technologies), IL-15 (R&D Systems), sVCAM-1 (R&D Systems), NGAL (BioPorto Diagnostics), and PIIANP (Merck). The ELISA-kits were used according to the manufacturer’s instructions. 

Interference with heterophilic human antibodies can occur especially when using sandwich ELISAs so that false-positive or false-negative results can arise [36]. In order to evaluate whether the values from our immunometric sandwich assays (NGAL, IL-15, and sVCAM1) were caused by heterophilic antibody interference, the serum of patients was also analyzed after pre-treatment with a heterophilic blocking tube (HBT, Scandibodies Laboratory). Application of HBT to our serum samples resulted in a 1.5-fold elevated serum level for IL-15, indicating non-specific antibody interference during the ELISA test. Considering this, our IL-15 values were not further evaluated.

### 2.4. Statistical Analysis 

For statistical analysis, GraphPadPrism5^®^ and R 3.4.2 [37] were used. Statistical significance was defined as *p*-values being equal or less than 0.05. The Wilcoxon signed-rank test was applied to evaluate whether biomarker levels quantified before and after exercise were different. The levels of biomarkers between the cohorts were compared using the Kruskal–Wallis test followed by Dunn’s post-hoc test. Spearman’s rank correlation coefficient was determined to describe any associations between clinical or radiological scores and the levels of biomarkers that might have been elevated in the OA group compared to controls. Data used to compare cohorts or to perform correlation analyses were obtained from the differences between the peak maxima of the biomarkers measured within 15 min of exercise and those measured 1 min before exercise. 

For the analysis of the biomarker time profiles we applied a nonparametric method for longitudinal data in factorial designs based on the marginal probabilistic index (PI) as described, e.g., in Acion et. al. [38] and Brunner et al. [39]. Our experimental setup was a so-called F1-LD-F1 design in the notation of the latter, and it was analyzed using the R package nparLD of Noguchi et al. [40]. The marginal PI in our design is the probability that a biomarker concentration in a cohort at a particular time point tends to be larger than a concentration in any cohort at any time point. Hence, a marginal PI of 50% indicates no tendency towards obtaining a biomarker concentration either larger or smaller than in any cohort at any time point investigated. The estimated values of the marginal PI for each cohort connected by polylines are presented along the time points of blood collection. Vertical segments show respective approximate pointwise 95% confidence intervals indicating a PI significantly different from 50% at a level of 5% if its interval does not contain the value 50%.

The biomarkers were also analyzed using the maximum concentrations of the individual time profiles as summarized values to derive the respective “summarized” PI to which we applied the methods of the PI for independent samples as described, e.g., in Konietschke et al. [41], and as implemented in the R package nparcomp of Konietschke et al. [42]. The estimated summary PIs together with their 95% confidence intervals are presented for all pairwise cohort comparisons as indicated on the horizontal axis. A 95% confidence interval of a summary PI not containing the value 50% indicates a significant difference between the respective cohorts on a level of 5%.

## 3. Results

### 3.1. Biomarker Levels

**Figure 1:**
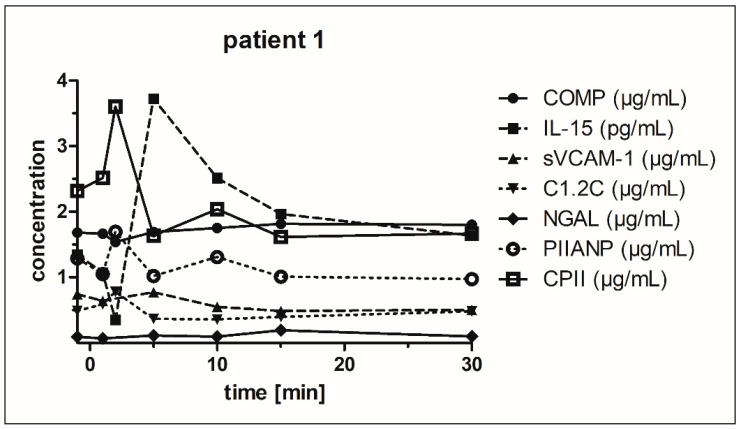
Time-dependent biomarker concentrations in the serum of a randomly selected osteoarthritis (OA) patient. The biomarker concentrations are shown before (t = −1 min, baseline) and after exercising of the hand and finger joints. The values at baseline are shown together with the y-axis at −1 min.

Figure 1 demonstrates in an exemplary manner that no common time point for sampling could be identified for all biomarkers after repeated forming of a load-controlled fist in the index hand. Nevertheless, mechanical exercise of the hand and finger joints appears to markedly increase the transport of biomarkers from the hand and finger joints to the venous blood of the corresponding forearm. In addition, proband-dependent differences with respect to both the level and the time point of the peak concentration of a biomarker were observed (Figure 1). 

Figure 1 shows that during the 60 min after exercise, incoming biomarkers only showed a maximum level during the first 15 min. There were only a few changes in biomarker concentrations at the subsequent sampling time points. Thus, the time points 45 and 60 min after exercise were not taken into account for further calculations. For the first time we found significantly increased levels of CPII, COMP, sVCAM-1, NGAL, and PIIANP during the first 15 min after exercise of the index hand within the venous blood of the ipsilateral forearm. However, levels of C1,2C did not change markedly after exercising of the index hand. 

**Figure 2:**
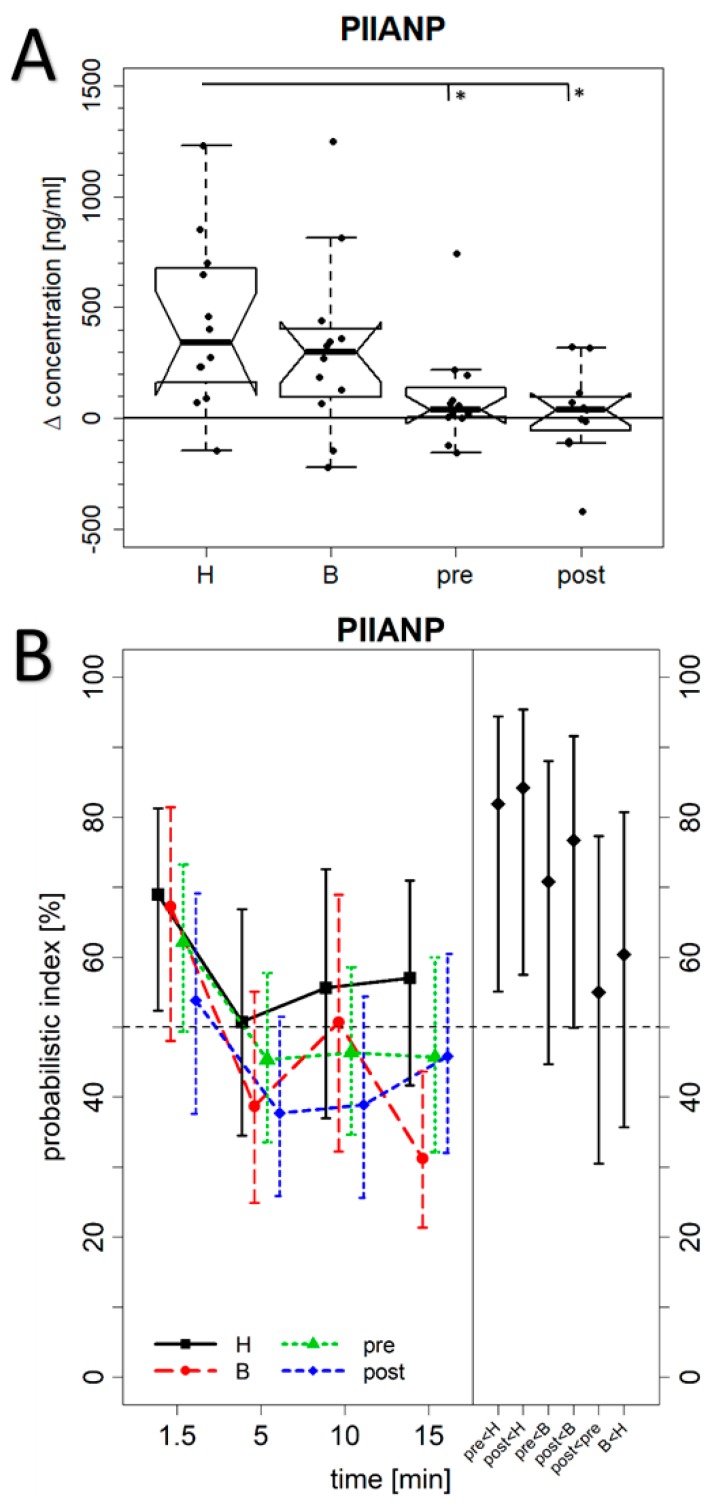
Serum concentration of PIIANP. (**A**) The difference between the maximum biomarker concentrations within the first 15 min after exercising of the hand and finger joints and the PIIANP level at baseline before exercising (H = cohort of patients with more Heberden-accentuated hand OA (*p* = 0.0024, *n* = 12); B = cohort of patients with more Bouchard-accentuated hand OA (*p* = 0.012, *n* = 12); pre = premenopausal control group (*p* = 0.09, *n* = 12); and post = postmenopausal control group (*p* = 0.64, *n* = 12)). Data are presented as notched boxplots with the X-axis at 0 indicating no altered concentration when compared with the time-point before exercising of the hand. The asterisks mark the statistically significant differences. (**B**) The estimated marginal probabilistic indices (PIs) of PIIANP concentrations for each cohort and time point (left, solid symbols connected by polylines of same type and color, augmented by pertaining pointwise 95% confidence intervals as vertical segments) and the estimated summarized PIs for all pairs of cohorts (right, black diamonds, with pairs of cohorts indicated on the horizontal axis) together with their pointwise 95% confidence intervals.

**Figure 3:**
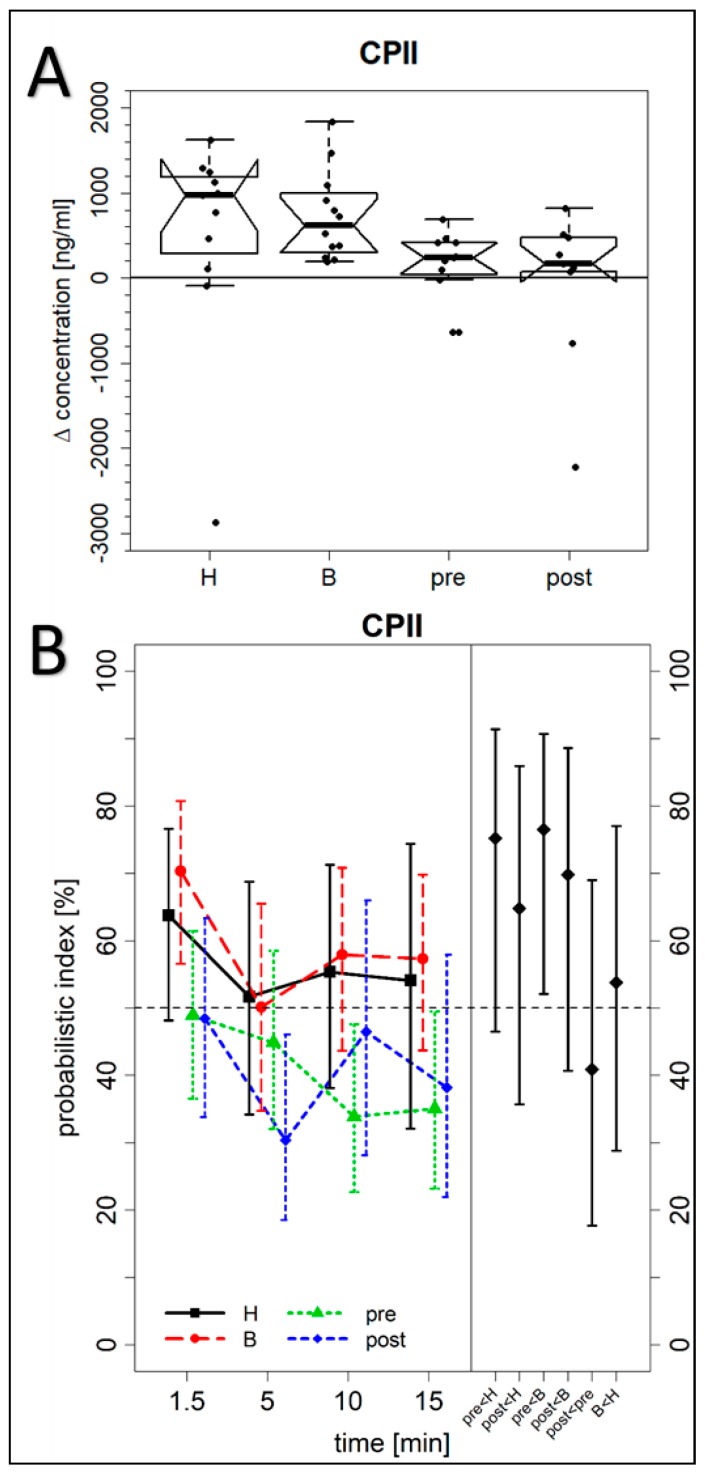
Serum concentrations of CPII. (**A**) The difference between the maximum biomarker concentration within the first 15 min after exercising of the hand and finger joints and the CPII level at baseline before exercising (H = cohort of patients with more Heberden-accentuated hand OA (*p* = 0.007, *n* = 12); B = cohort of patients with more Bouchard-accentuated hand OA (*p* = 0.005, *n* = 12); pre = premenopausal control group (*p* = 0.28, *n* = 12); and post = postmenopausal control group (*p* = 0.50, *n* = 12)). For further details see the caption of Figure 2A. (**B**) Fully analogous to Figure 2B, but here for CPII concentrations.

**Figure 4:**
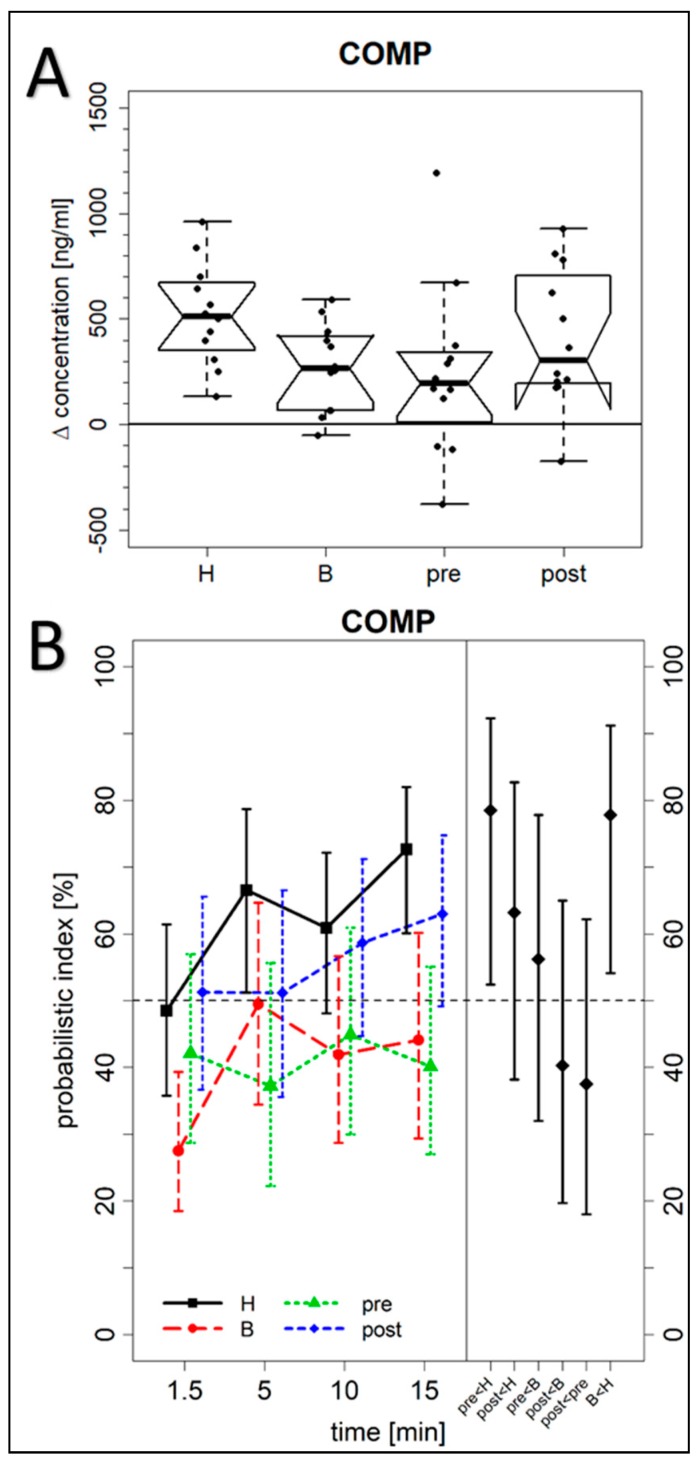
Serum concentrations of COMP. (**A**) The difference between the maximum biomarker concentrations within the first 15 min after exercising of the hand and finger joints and the COMP level at baseline before exercising were calculated (H = cohort of patients with more Heberden-accentuated hand OA (*p* = 0.0005; *n* = 12); B = cohort of patients with more Bouchard-accentuated hand OA (*p* = 0,0042; *n* = 12); pre = premenopausal control group (*p* = 0.04; *n* = 12); and post = postmenopausal control group (*p* = 0.001; *n* = 12)). For further details see the caption of Figure 2A. (**B**) Fully analogous to Figure 2B, but here for COMP concentrations.

An additional important aim of our study was to investigate whether differences exist between the biomarker levels found in the OA cohort versus controls. We calculated for each biomarker the intra-individual differences between their maximum concentrations determined within the first 15 min after exercise and their baseline values (Table 2) as analyzed 1 min before exercise of the index hand. Figure 2 demonstrates marked differences between the OA groups and the pre- or postmenopausal control group for PIIANP as determined with the Kruskal–Wallis test (*p* = 0.007). PIIANP was significantly higher in the Heberden’s OA cohort as compared to the two pre- and postmenopausal control groups (Dunn test, *p* = 0.050 and *p* = 0.015, respectively). However, we could not find any significant differences between the four different cohorts for NGAL, CPII, COMP, C1,2C, and sVCAM1 (Figure 3: CPII; Figure 4: COMP; Figure S1–S3: NGAL, C1,2C, and sVCAM1).

### 3.2. Probabilistic Index

For PIIANP (Figure 2B, left), the analysis of time-by-cohort interactions of the marginal PI did not reveal any significant differences between any two cohorts, i.e., the time trends of concentration levels were not significantly different between any two cohorts. Likewise, the main cohort effects did not reveal any significant differences between any two of the four cohorts, which means that the concentration levels over all time points in any cohort tend towards similar values compared to those of any other cohort. As an example, to illustrate how to read the figure, look at the time point 1.5 min where there is an estimated marginal PI of 69% for the PIIANP concentration in the cohort with the Heberden-accentuated hand OA shown. This marginal PI means that with a probability of 69% a biomarker concentration in this cohort at this time is larger than a PIIANP-concentration in any cohort at any time. This estimated PI has an approximate 95% confidence interval from 52% to 81%.

The summarized PI of the Heberden’s OA cohort relative to the premenopausal control cohort (Figure 2B, right, “pre < H”) is estimated to be 82%, meaning that with a probability of 82% a maximum biomarker concentration in the cohort with Heberden’s OA is larger than a maximum concentration in the premenopausal control cohort. This PI is associated with a *p*-value of 0.023, revealing a significant difference between the premenopausal control and the Heberden’s OA cohort. The PI for Heberden’s OA vs. the postmenopausal control cohort (Figure 2B, right, “post < H”) is 84% (*p* = 0.017), while it is 55% (*p* = 0.70) for pre vs. post (Figure 2B, right, “post < pre”). These data demonstrate large differences in tendency between the Heberden’s OA and the pre- or postmenopausal control cohorts, respectively, but almost no difference in tendency between both control cohorts.

For CPII (Figure 3B, left) the analysis of time-by-cohort interactions of the marginal PI did not reveal any significant difference between any two cohorts, i.e., the time trends of concentration levels were not significantly different between any two cohorts. However, the main cohort effects did reveal a significant difference between the Bouchard-accentuated hand OA and the premenopausal control cohort (*p* = 0.028), but not between any other two cohorts. This means that the concentration levels over all time points in Bouchard’s OA tend to higher values than in the premenopausal control cohort.

The summarized PI of the Bouchard’s OA cohort relative to the premenopausal control cohort (Figure 3B, right, “pre < B”) shows that with a probability estimated at 76% (*p* = 0.035), a maximum biomarker concentration in the Bouchard’s OA cohort was larger than a maximum concentration in the premenopausal control cohort. The PI for Bouchard’s OA vs. postmenopausal control cohort (Figure 3B, right, “post < B”) is 70% (*p* = 0.18), and 41% (*p* = 0.54) for premenopausal vs. postmenopausal cohorts (Figure 3B, right, “post < pre”). These data demonstrate a large and significant difference in tendency between Bouchard’s OA and the premenopausal control cohort, a large but not significant difference between Bouchard’s OA and the postmenopausal control cohort, but almost no difference between the two control cohorts.

For COMP (Figure 4B, left), the analysis of time-by-cohort interactions of the marginal PI did not reveal any significant difference between any two cohorts, i.e., the time trends of concentration levels were not significantly different between any two cohorts. However, the main cohort effects did reveal a significant difference between Heberden’s and Bouchard’s OA cohorts (*p* = 0.004), between Heberden’s OA and the premenopausal control cohort (*p* = 0.018), but not between any other two cohorts. This means that the concentration levels over all time points in Heberden’s OA tend towards higher values than is the case in Bouchard’s OA and in the premenopausal control cohort.

The summarized PI of the Heberden’s OA cohort relative to the premenopausal control cohort (Figure 4B, right, “pre < H”) showed that with a probability estimated at 78% (*p* = 0.034), a maximum biomarker concentration in the Heberden’s OA cohort was larger than the maximum concentration in the premenopausal control cohort. The PI for Heberden’s OA vs. Bouchard’s OA (Figure 4B, right, “B < H”) is 78% (*p* = 0.024), and 37% (*p* = 0.32) for the premenopausal vs. the postmenopausal control cohort (Figure 4B, right, “post < pre”). This demonstrates large and significant differences between the Heberden’s OA cohort and the premenopausal control and Bouchard’s OA cohort, respectively, but no significant difference between the two control cohorts.

### 3.3. Correlation of Biomarkers with X-ray Scores

The correlation analysis of the biomarker peaks after exercising the OA index hand using the radiological KL and Kallman scores was performed by Spearman’s rank correlation. For all biomarkers, no significant correlations between the maximum serum concentrations of the OA cohort and the single, total, or average scores of the radiographic examinations could be determined. Furthermore, a correlation was calculated between the biomarker levels and the numbers of radiologically affected joints being defined as having a KL score [7,35] of at least two. However, no marked correlation between the number of radiologically diagnosed joints and biomarker levels was found.

### 3.4. Correlation of Biomarkers with Clinical Scores

Spearman’s rank correlation was used to determine possible correlations between clinical scores obtained from questionnaires and the maximum levels of biomarkers found to be significantly elevated in OA cohorts versus controls. The total scores of the individual clinical scores, as well as the scores from individual subsections for pain, stiffness, and hand function were included. However, PIIANP levels did not correlate significantly with the results of any of the questionnaires including the AUSCAN™, HAQ, and VAS pain scores, nor the radiological Kallman score.

## 4. Discussion

Many OA biomarker studies have focused on larger joints such as knees or hips, because higher levels of biomarkers are produced by those joints, which are then easier to analyze. Therefore, the aim of our exploratory study was to obtain elevated levels of biomarkers after a standardized and load-controlled mechanical exercise of the OA-affected hand and finger joints amongst patients as opposed to healthy controls.

Most remarkably, a significantly elevated PIIANP level was determined in patients with a more Heberden-accentuated hand OA compared with healthy controls. In addition, the summarized PI, which uses the maximum concentrations of the individual time-dependent profiles, also revealed that PIIANP tends with a significantly elevated probability towards larger values in Heberden-accentuated hand OA cohort than in both control cohorts. PIIANP is a marker for cartilage synthesis, and the elevated level indicates a compensatory up-regulated repair of cartilage. Our data are corroborated by the lower PIIANP levels seen in healthy volunteers after exercise of their index hand due to their intact cartilage homeostasis. However, PIIANP was reported to be present at slightly reduced serum levels in patients with hand [18] and advanced knee OA [43,44,45], indicating that there are cohort specific differences.

Another remarkable finding was that CPII might be able to distinguish between Bouchard-accentuated hand OA and premenopausal controls. Furthermore, our explorative study indicates that COMP may be able to discriminate between both subtypes of hand OA as well as between Heberden’s OA and premenopausal controls. In addition, our calculations of the marginal PI suggest that CPII, PIIANP, and COMP can be used to distinguish between various cohorts. However, we were not able to detect altered COMP or sVCAM-1 levels in hand OA compared to healthy controls. Such data is consistent with the elevated biomarker concentrations reported by others [16,18], which may be due to our approach of considering contributions from other joints by measuring background levels [46]. In contrast to the data we obtained from clearly defined female cohorts, Kalichman et al. [47] reported a positive correlation of sVCAM-1 with the number of OA affected hand joints in a cross-sectional population-based study. Currently, we are not aware of any other studies addressing IL-15 and NGAL as possible biomarkers for hand OA.

A few studies have investigated the concentration profiles of a number of biomarkers after exercising of the respective joints [29,30,31,32]. For instance, Frisbie et al. found an increased level of CPII in the serum of horses after training for several days [32]. Other studies assessing knee OA patients [29], physically active healthy adults [30], and marathon runners [31] reported a maximum concentration of COMP at the earliest 30 min after exercise. The increase in serum biomarker levels after exercise may be derived from the pressure and shear forces generated within the joint during exercise which in turn might induce an elevated “clearance” of the synovia. Furthermore, exercise might stimulate the circulation so that lymphatic drainage from the respective joints may be accelerated.

Our study design involving a precisely defined blood sampling time enabled us to establish a kinetic biomarker level curve directly after exercise showing a maximum biomarker concentration during the first 15 min after mechanical exercise. We also determined the background levels of each biomarker one minute before mechanical exercising of the hand. This background level reflects contributions from other joints, since we cannot exclude that any subclinical cartilage alterations may have taken place given that radiological or magnet-resonance-imaging had not been carried out.

The limitations of our study include the observation that 11 out of 24 OA patients suffered an erosive OA (EOA) in at least one joint of the hand as determined radiographically. Published biomarker studies sometimes subcategorize hand-OA into EOA and non-EOA [14,15,19,48]. EOA is characterized by joint inflammation and marked, radiologically detectable joint destruction [48,49]. The results of our exploratory study were not predictable at the onset. For this reason, the planning of the sample size for statistically analyzing EOA versus non-EOA subgroups proved to be inadequate.

## 5. Conclusions

The present study shows for the first time that elevated levels of serum biomarkers are detectable within 15 min after mechanical exercising of the hand and finger joints. Remarkably, PIIANP was found to be markedly elevated in the serum of patients with a more Heberden-accentuated hand OA whereas COMP may prove useful for distinguishing between the two subtypes of hand OA. The results of our exploratory study serve as a basis for further studies identifying serum biomarkers for the diagnosis, staging, and therapeutic monitoring of both early and advanced stages of OA in the hand and finger joints [50].

## Figures and Tables

**Table 1:** Characterization of the study cohorts.

	Heberden’s OA	Bouchard’s OA	Pre-Group	Post-Group
***n***	12	12	12	12
**age**	60.3 ± 5.7 60.0 (55.8–63.8)	62.8 ± 7.1 63.5 (56.0–68.0)	23.4 ± 1.2 *** 23.0 (23.0–24.8)	59.9 ± 7.0 61.0 (52.0–66.5)
**BMI** (kg/m²)	26.0 ± 3.4 25.6 (23.1–28.5)	26.5 ± 3.4 26.8 (22.8–29.0)	21.5 ± 2.0 *** 21.0 (20.0–22.8)	23.0 ± 3.3 22.9 (20.8–23.9)
**CRP** (mg/dL)	3.5 ± 0.5 3.3 (3.1–3.9)	5.0 ± 2.2 4.1 (3.4–7.5)	2.2 ± 2.3 1.4 (0.4–3.1)	1.0 ± 0.9 *** 0.7 (0.4–1.6)
**AUSCAN total** (0–1500 mm)	632 ± 335 735 (247–933)	643 ± 388 542 (256–1016)	0.0 ± 0.0 *** 0.0 (0.0–0.0)	5.5 ± 12.9 *** 0.0 (0.0–0.0)
**AUSCAN stiffness** (0–100 mm)	37.8 ± 30.2 33.5 (12.3–63.8)	41.8 ± 28.3 39.0 (17.8–62.5)	0.0 ± 0.0 *** 0.0 (0.0–0.0)	2.6 ± 9.0 *** 0.0 (0.0–0.0)
**AUSCAN pain** (0–500 mm)	198 ± 137 186 (79.5–303)	190 ± 121 180 (73.0–292)	0.0 ± 0.0 *** 0.0 (0.0–0.0)	0.0 ± 0.0 *** 0.0 (0.0–0.0)
**AUSCAN ADL** (0–900 mm)	397 ± 211 421 (150–541)	411 ± 270 335 (119–701)	0.0 ± 0.0 *** 0.0 (0.0–0.0)	2.9 ± 10.1 *** 0.0 (0.0–0.0)
**VAS-pain** (0–100 mm)	39.6 ± 27.4 37.1 (15.9–60.5)	38.0 ± 24.2 35.9 (14.6–58.4)	0.0 ± 0.0 *** 0.0 (0.0–0.0)	0.0 ± 0.0 *** 0.0 (0.0–0.0)
**HAQ** (0–3)	0.7 ± 0.5 0.6 (0.3–1.0)	0.9 ± 0.7 0.8 (0.2–1.7)	0.0 ± 0.0 *** 0.0 (0.0–0.0)	0.0 ± 0.1 *** 0.0 (0.0–0.0)
**KL score** **total**	23.6 ± 7.4 24.0 (16.5–30.8)	27.4 ± 10.7 30.5 (22.8–33.8)	n.d.	n.d.
**Kallman** **total**	32.8 ± 11.0 34.5 (28.3–40.8)	36.1 ± 16.3 39.0 (28.8–46.8)	n.d.	n.d.

Data shown represent means ± standard deviations and medians with interquartile ranges in brackets. Data of both OA cohorts are statistically significant different from those of the pre- and/or postmenopausal control group according to the Kruskal–Wallis test followed by Dunn’s post-hoc test or the Wilcoxon signed-rank test: *** *p* < 0.001, n.d. = not determined. Pre-group = premenopausal control group; Post-group = postmenopausal control group; AUSCAN = Australian/Canadian Hand Osteoarthritis Index; ADL = activity of daily living; KL = Kellgren/Lawrence; VAS = visual analogue scale; HAQ = Health Assessment Questionnaire.

**Table 2:** Baseline biomarker levels.

Biomarker	Heberden’s OA	Bouchard’s OA	Pre-Group	Post-Group	*p*-Value
**CPII** (µg/mL)	2.0 ± 1.0 1.9 (1.4–2.5)	1.5 ± 0.6 1.6 (0.9–2.0)	1.6 ± 0.8 1.8 (0.7–2.1)	1.9 ± 1.4 2.0 (0.8–3.0)	0.1372
**C1,2C** (ng/mL)	691 ± 287 621 (499–910)	605 ± 164 571 (524–711)	556 ± 144 549 (471–595)	931 ± 1087 586 (519–822)	0.4890
**COMP** (µg/mL)	1.8 ± 0.4 1.8 (1.6–2.0)	2.0 ± 0.3 2.0 (1.8–2.3)	1.2 ± 0.3 1.1 (1.0–1.5)	1.7 ± 0.4 1.6 (1.3–2.1)	0.0605
**sVCAM1** (ng/mL)	522 ± 288 462 (272–669)	485 ± 251 497 (289–700)	585 ± 220 627 (404–699)	587 ± 136 588 (512–686)	0.2690
**NGAL** (ng/mL)	100 ± 52.0 97.5 (56.9–126)	113 ± 32.7 107 (92.9–127)	120 ± 58.2 108 (79.8–125)	128 ± 72.5 102 (73.0–161)	0.5063
**PIIANP** (ng/mL)	1394 ± 383 1285 (1163–1609)	1389 ± 487 1210 (1059–1791)	1027 ± 294 893 (830–1216)	1172 ± 386 1029 (946–1353)	0.0070

Data shown represent the means ± standard deviations, medians with interquartile ranges in brackets, and the *p*-values of the Kruskal–Wallis test. Pre-group = premenopausal control group, Post-group = postmenopausal control group.

## Supplementary Materials

The following are available online at https://www.mdpi.com/2077-0383/8/10/1545/s1.
