# Supplementary material for: Biomarkers of Hand Osteoarthritis Are Detectable after Mechanical Exercise
Source: J Clin Med. 2019 Sep 26;8(10):1545. doi: 10.3390/jcm8101545 (PMC6832610; doi:10.3390/jcm8101545)
Supplement: Supplementary file 1 [file jcm-08-01545-s001.pdf]

Supplementary Material

Biomarkers of Hand Osteoarthritis are Detectable after Mechanical Exercise

Bender A, Kaesser U, Eichner G, Bachmann G and Steinmeyer J

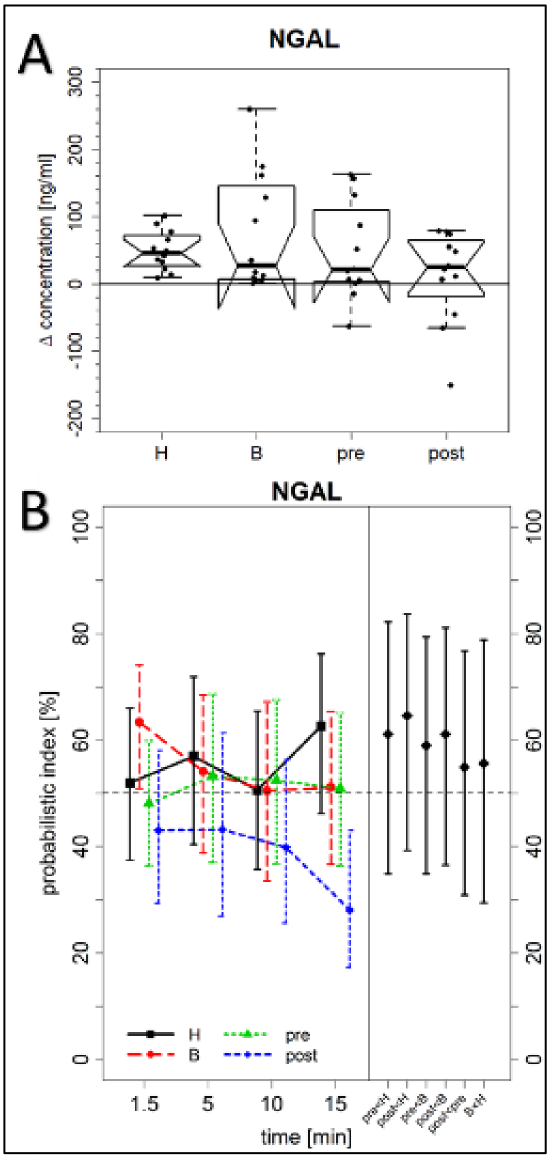

Figure S1. Serum concentrations of NGAL. **(A)** The difference between the maximum biomarker concentration within the first 15 minutes after exercising of the hand and finger joints and the NGAL level at baseline before exercising [H = cohort of patients with more Heberden-accentuated hand OA (p = 0.0005, n = 12), B = cohort of patients with more Bouchard-accentuated hand OA (p = 0.005, n = 12); pre = premenopausal control group (p = 0.03, n = 12); post = postmenopausal control group (p = 0.30, n = 12)]. For further details see caption of Figure 2A. **(B)** Fully analogous to Figure 2B, but here for NGAL concentrations.

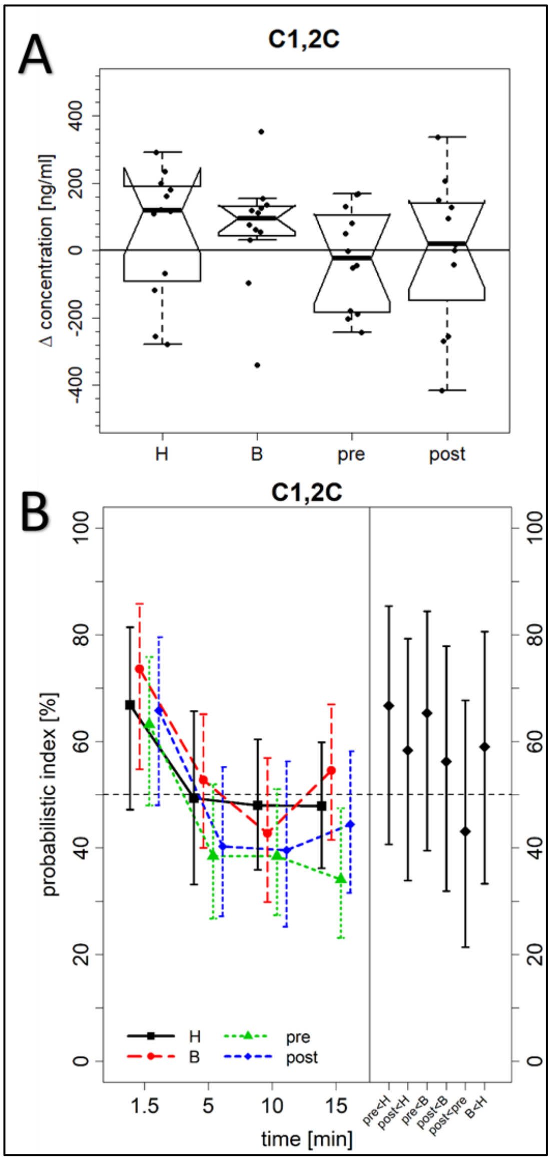

Figure S2. Serum concentrations of C1,2C. **(A)** The difference between the maximum biomarker concentration within the first 15 minutes after exercising of the hand and finger joints and the C1,2C level at baseline before exercising [H = cohort of patients with more Heberden-accentuated hand OA (p = 0.34, n = 12), B = cohort of patients with more Bouchard-accentuated hand OA (p = 0.08, n = 12); pre = premenopausal control group (p = 0.47, n = 12); post = postmenopausal control group (p = 0.90, n = 12)]. For further details see caption of Figure 2A. **(B)** Fully analogous to Figure 2B, but here for C1,2C concentrations.

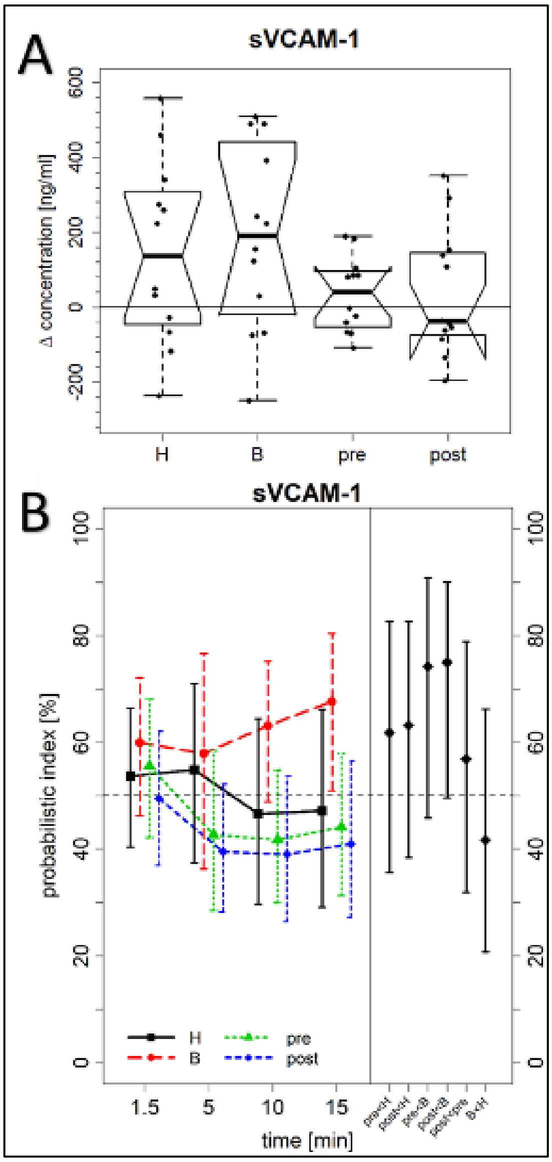

Figure S3. Serum concentrations of sVCAM1. **(A)** The difference between the maximum biomarker concentration within the first 15 min after exercising of the hand and finger joints and the sVCAM1 level at baseline before exercising [H = cohort of patients with more Heberden-accentuated hand OA ($p = 0.04$, $n = 12$), B = cohort of patients with more Bouchard-accentuated hand OA ($p = 0.09$, $n = 12$); pre = premenopausal control group ($p = 0.30$, $n = 12$); post = postmenopausal control group ($p = 0.62$, $n = 12$)]. For further details see caption of Figure 2A. **(B)** Fully analogous to Figure 2B, but here for sVCAM1 concentrations.
